# Supplementary material for: A persistent variant telomere sequence in a human pedigree
Source: Nat Commun. 2024 Jun 1;15:4681. doi: 10.1038/s41467-024-49072-9 (PMC11144197; doi:10.1038/s41467-024-49072-9)
Supplement: Supplementary file 1 — Supplementary Information [file 41467_2024_49072_MOESM1_ESM.pdf]

A persistent variant telomere sequence in a human pedigree

Angela M. Hinchie[1,2], Samantha L. Sanford[3,4], Kelly E. Loughridge[1,2], Rachel M. Sutton[1,2], Anishka H. Parikh[1,2], Agustin A. Gil Silva[1,2], Daniel I. Sullivan[1,2], Pattra Chun-On[1,2], Matthew R. Morrell[2], John F. McDyer[2], Patricia L. Opresko[3,4,5], Jonathan K. Alder[1,2]*

Affiliations:

[1]Dorothy P. and Richard P. Simmons Center for Interstitial Lung Disease, Pittsburgh, PA, USA.

[2]Division of Pulmonary, Allergy, and Critical Care Medicine; Pittsburgh, PA, USA.

[3]Environmental and Occupational Health Department, School of Public Health, University of Pittsburgh; Pittsburgh, PA, USA.

[4]University of Pittsburgh Medical Center, Hillman Cancer Center, Pittsburgh, PA, USA.

[5]Pharmacology and Chemical Biology Department, University of Pittsburgh School of Medicine, PA, USA.

*Corresponding author. Email: jalder@pitt.edu

Supplementary Data

Supplementary Tables
Supplementary Figures

Tables

Supplementary Table 1. Primers used for reported studies.

PRIMER NAME	SEQUENCE (5` TO 3`)
DIRECT ASSAY PRIMER 1 (WT)	GGTTAGGGTTAGGGTTAG
DIRECT ASSAY PRIMER 2 (C50A)	GGTTAGGGTTAGTTTAG
DIRECT ASSAY PRIMER A5	TTAGGGTTAG<u>C</u>GTTAGGG
EMSA WT	/Cy5.5/GGTTAGGGTTAG
EMSA VAR	/Cy5.5/G<u>T</u>TTAGG<u>T</u>TTAG
EMSA G TO C	/Cy5.5/GGTTAG<u>C</u>GTTAG
TEL END SEQ REPAIR – NEXTERA MM	GTC TCG TGG GCT CGG AGA TGT GTA TAA GAG ACA GGG GGG GGG GGG GMM
TEL END SEQ REPAIR - NEXTERA HW	GTC TCG TGG GCT CGG AGA TGT GTA TAA GAG ACA GGG GGG GGG GGG GHW
TEL END SEQ PCR1 FWD NEXTERA 1-1	TCG TCG GCA GCG TCA GAT GTG TAT AAG AGA CAG NNN NNG KTT AGG KTT AGG KTT AGG KTT AG
TEL END SEQ PCR1 FWD NEXTERA 1-2	TCG TCG GCA GCG TCA GAT GTG TAT AAG AGA CAG NNN NNN GKT TAG GKT TAG GKT TAG GKT TAG
TEL END SEQ PCR1 REV NEXTERA 2	GTC TCG TGG GCT CGG AGA TG
TEL END SEQ PCR2 FWD S502	AAT GAT ACG GCG ACC ACC GAG ATC TAC ACC TCT CTA TTC GTC GGC AGC GTC
TEL END SEQ PCR2 FWD S503	AAT GAT ACG GCG ACC ACC GAG ATC TAC ACT ATC CTC TTC GTC GGC AGC GTC
TEL END SEQ PCR2 REV N701	CAA GCA GAA GAC GGC ATA CGA GAT TAA GGC GAG TCT CGT GGG CTC GG
TEL END SEQ PCR2 REV N702	CAA GCA GAA GAC GGC ATA CGA GAT CGT ACT AGG TCT CGT GGG CTC GG

Supplementary Table 2. PNA Probes used.

Probe Name	SEQUENCE (5` TO 3`)
TelC-Cy3	/Cy3/$(CCCTAA)_3$
TTAGGT – C probe – Alexa647	/Alexa647/$(ACCTAA)_3$
CENPB-Alexa488	/Alexa488/ATTCGTTGGAAACGGGA

Supplementary Figures

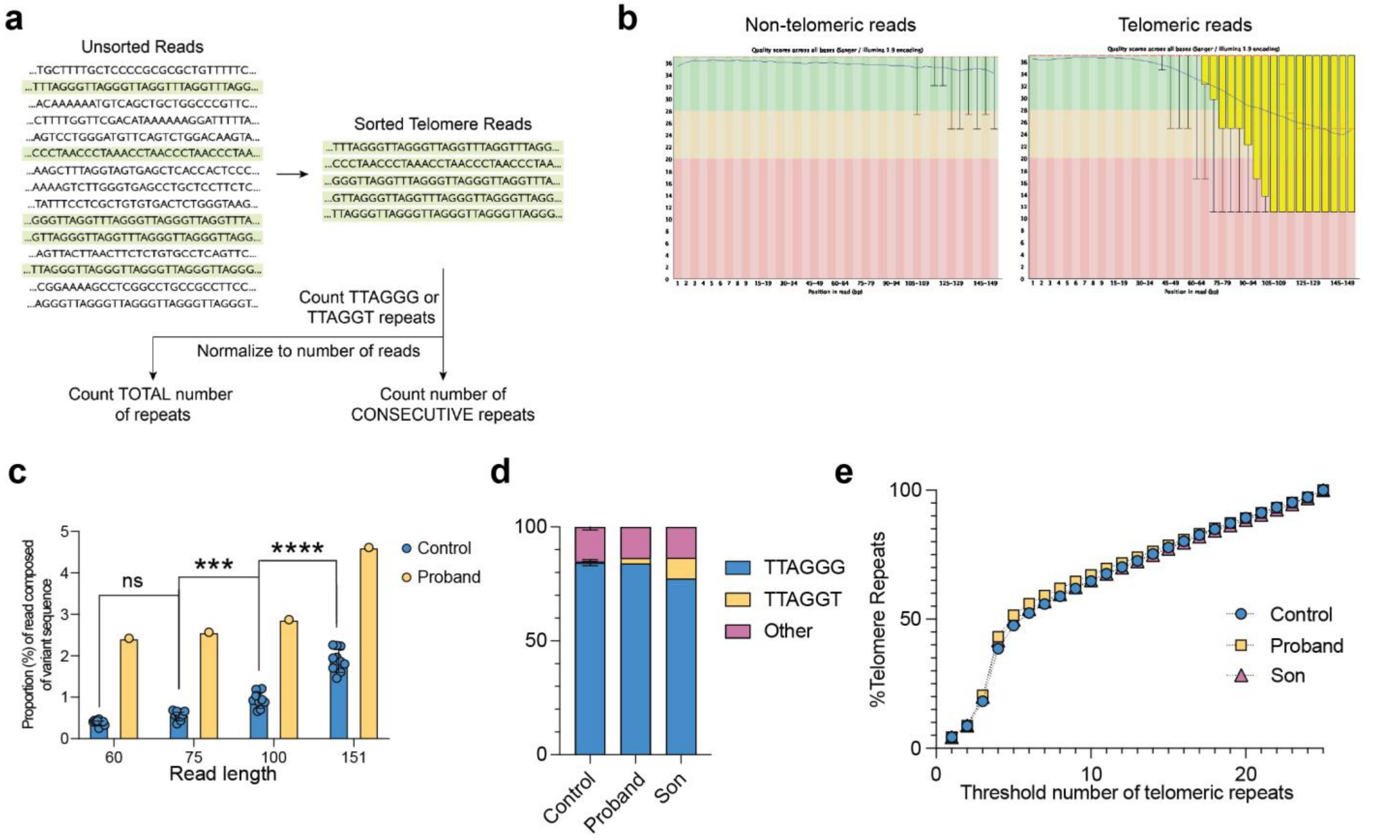

Supplementary Figure 1. Analysis of *in vivo* variant telomere sequence incorporation. a) Schematic showing the methodology of extracting and counting telomeric repeats. **b)** Read quality scores for 1000 random non-telomeric reads versus telomeric reads from the proband measured by the FastQC software (https://www.bioinformatics.babraham.ac.uk/projects/fastqc/). **c)** Sequencing reads were 151 base pairs long. The bar graph shows the percentage of variant repeats per total sequence when base pairs from the lower quality 3` end were removed. Mean±s.d. is shown; groups were compared with one-way ANOVA and Tukey's multiple comparison. **d)** Percentage of wildtype, variant, and other telomere repeats in controls, the proband, and his son. **e)** The proportion of total DNA sequence that is composed of telomere repeats is a function of the threshold that is used to deem a read telomeric. We arbitrarily selected a threshold of 13 repeats (~50%; GGTTAG or GTTTAG) for a read to be deemed telomeric. Telomeric reads were further trimmed to 60 bps for our analysis. Ns, nonsignificant p>0.05, ****p* <0.001, *****p* <0.0001. Source data are provided as a Source Data file.

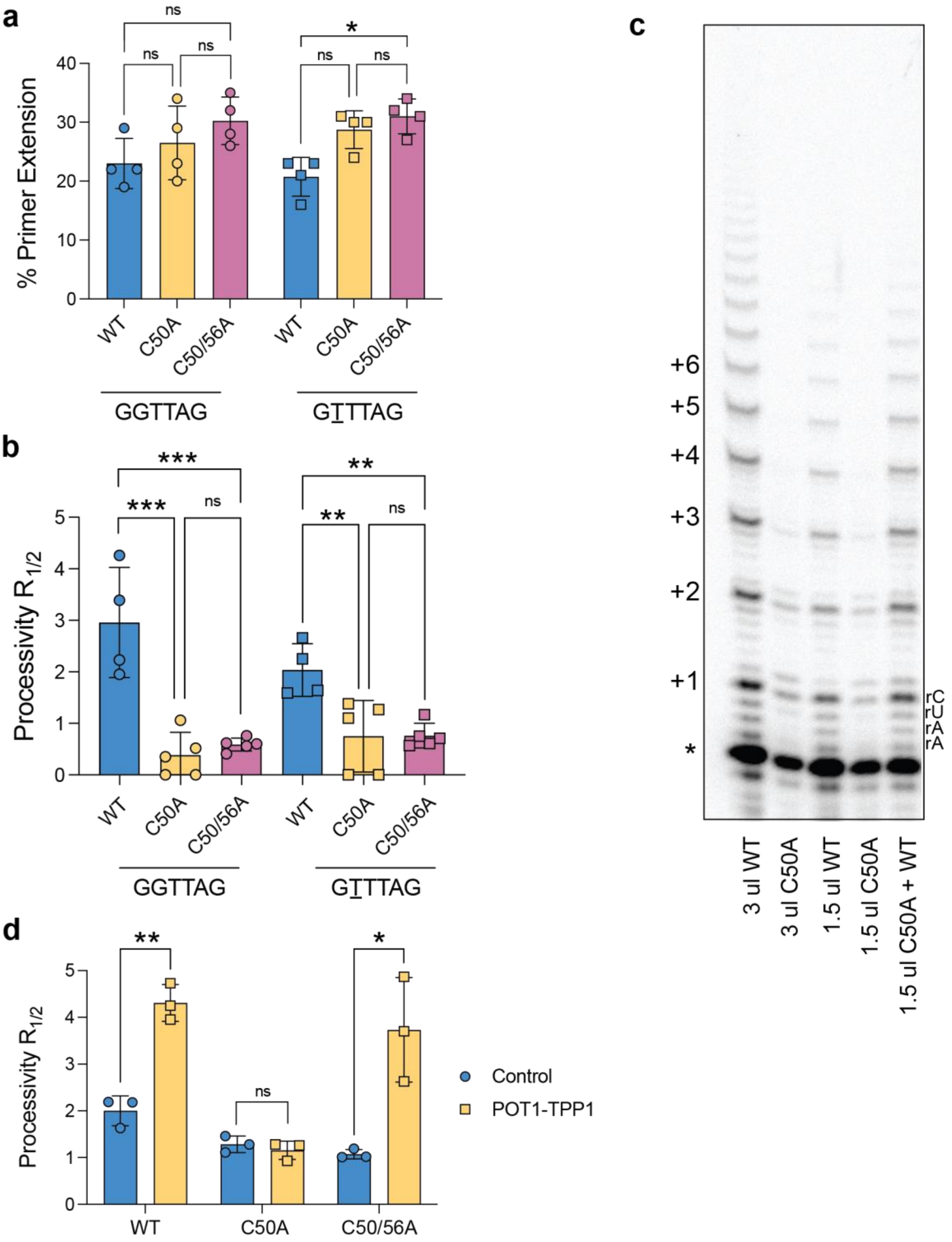

Supplementary Figure 2. Biochemical analysis of extension and processivity. a) Percent of primer extension by WT, C50A, and C50/56A TR in the direct assay from Fig. 2c, n=4 biological replicates, mean±s.d., one-way ANOVA with Tukey's multiple comparison correction. **b)** Traditional method of processivity quantitation from Fig. 2c, WT (n=4), C50A (n=5), C50/56a (n=5) biological replicates, one-way analysis of variance (ANOVA) with Tukey's multiple comparison. **c)** Telomerase direct assay mixing WT and C50A TR in equal ratios from reaction with primer (TTAGGG)$_3$. * indicates the position of the radiolabelled primer and the numbers on the left side indicate the number of added repeats. **d)** Processivity quantification of (2d), processivity with POT1-

TPP1 addition, n=3 biological replicates, mean±s.d., two-tailed unpaired t-test. ns, non-significant, $p \geq 0.05$, *$p < 0.05$, **$p < 0.01$, ***$p < 0.001$. Source data are provided as a Source Data file.

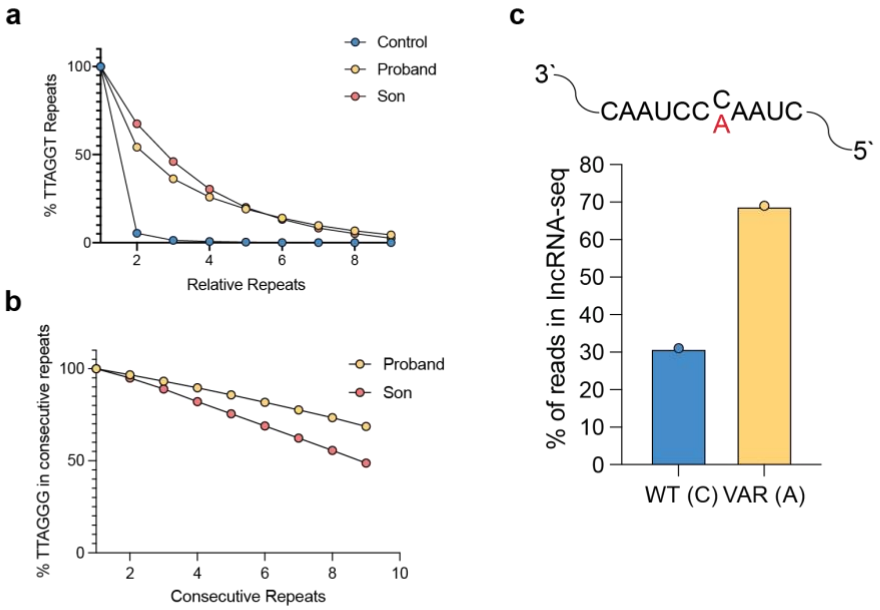

Supplementary Figure 3. *In vivo* processivity measurement. a) Proportion of TTAGGT repeats found in consecutive repeats for the proband, his son, and the 10 controls. **b)** Percentage of TTAGGG repeats found in n or more consecutive repeats for the proband and his son. **c)** Proportion of reads mapping to the wild type and variant alleles from long non-coding RNA-sequencing (lncRNA-seq) data derived from the probands PBMC's. Source data are provided as a Source Data file.

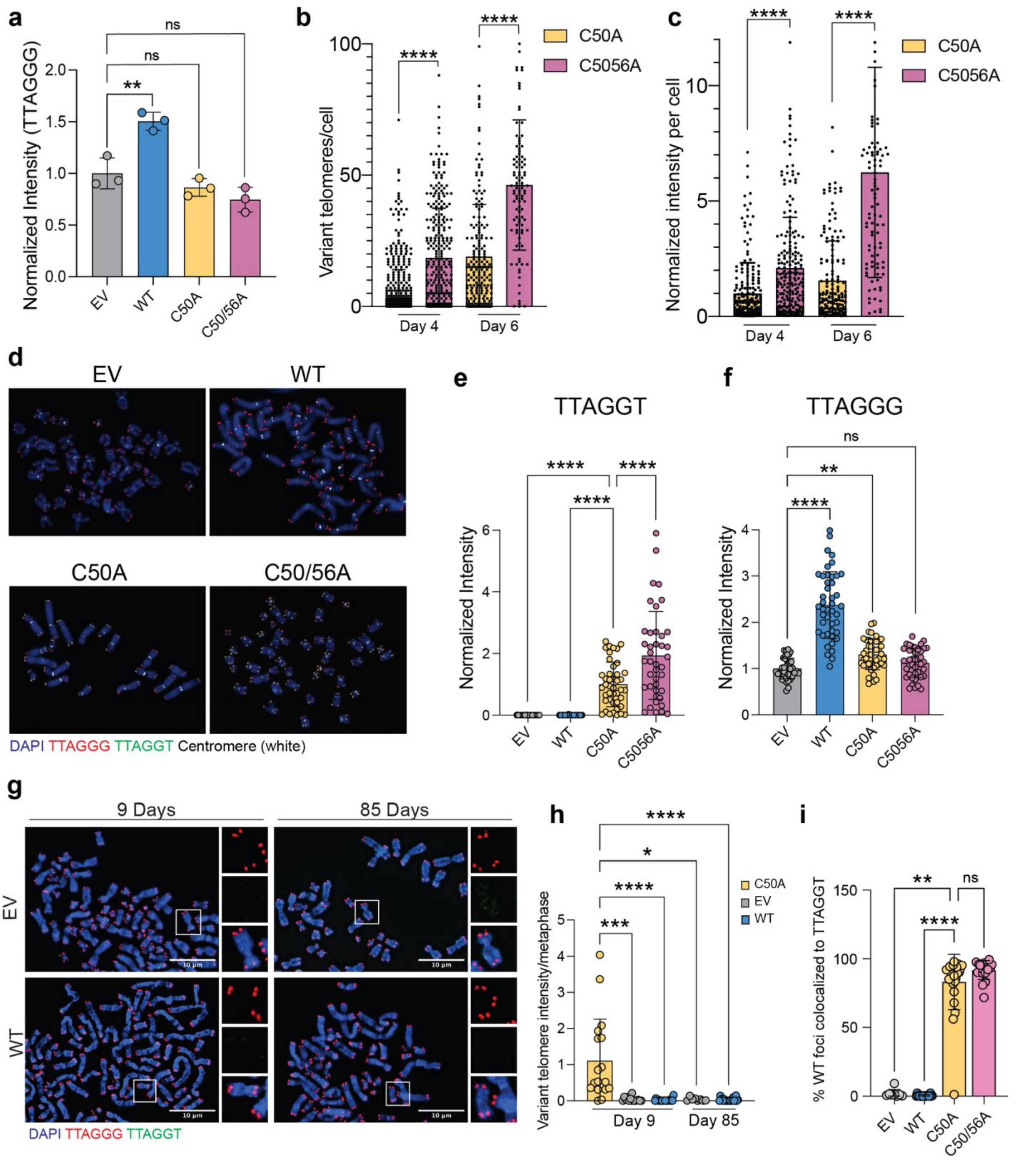

Supplementary Figure 4: Analysis of *in vivo* variant telomere addition. a) Intensity of TTAGGG telomere foci in hTERT-RPE cells 6 days post transduction with EV, WT, C50A or C50/56A TR, normalized to EV. Mean±s.d. is shown; groups were compared with one-way ANOVA and Dunnet's Multiple Comparison. **b)** Number of variant telomere foci per nucleus in LOX Melanoma cells transduced with C50A or C50/56A TR at 4- or 6-days post-transduction. **c)** Intensity of variant (TTAGGT) telomere foci in LOX Melanoma cells

transduced with C50A or C50/56A TR at 4- or 6-days post-transduction, normalized to C50A day 4. For (b) and (d), data points are individual nuclei, C50A Day 4 (283), C50/56A Day 4 (289), C50A Day 6 (188), C50/56A Day 6 (106). median±s.d. is shown; groups compared by two-tailed unpaired t-test. **d)** Representative photomicrograph of metaphase spreads of HCT116 cells transduced with hTERT and either EV, WT, C50A, or C50/56A TR**,** normalized to EV intensity. **e)** Quantification of variant telomere intensity from (d), normalized to C50A intensity. **f)** Quantification of wildtype telomere intensity from (d), normalized to EV intensity. For (e) and (f)**,** EV (n=40), WT (n=44), C50A (n=43), C50/56A (n=41), median±s.d. is shown; groups are compared with one-way ANOVA with Dunnet's Multiple Comparison. **g)** Representative photomicrograph of metaphase FISH of LOX Melanoma cells with the wildtype and variant sequence at 9- and 85-days post-transduction with EV or WT TR. **h)** Variant telomere intensity per metaphase from (g), normalized to C50A day 9 from Fig. 2f **i)** Percentage of wildtype telomeres co-localized with a variant telomere focus at day 85 from Fig. 2f. (h) and (i) compared by Kruskal-Wallis test with Dunn's multiple comparison. For all comparisons, ns, non-significant, $p \geq 0.05$, *$p < 0.05$, **$p < 0.01$, ***$p < 0.001$, ****$p < 0.0001$. Source data are provided as a Source Data file.

a

Variant	Designation	Template 3'-5'	Telomere
Wildtype	WT	CAAUCCCAAUC	TTAGGG
Patient Variant	C50A	CAAUCCAAAUC	TTAGGT
Complementary Variant	C50/56A	AAAUCCAAAUC	TTAGGT
Toxic Variant	AU5	AAUAUAUAUAU	TATATA

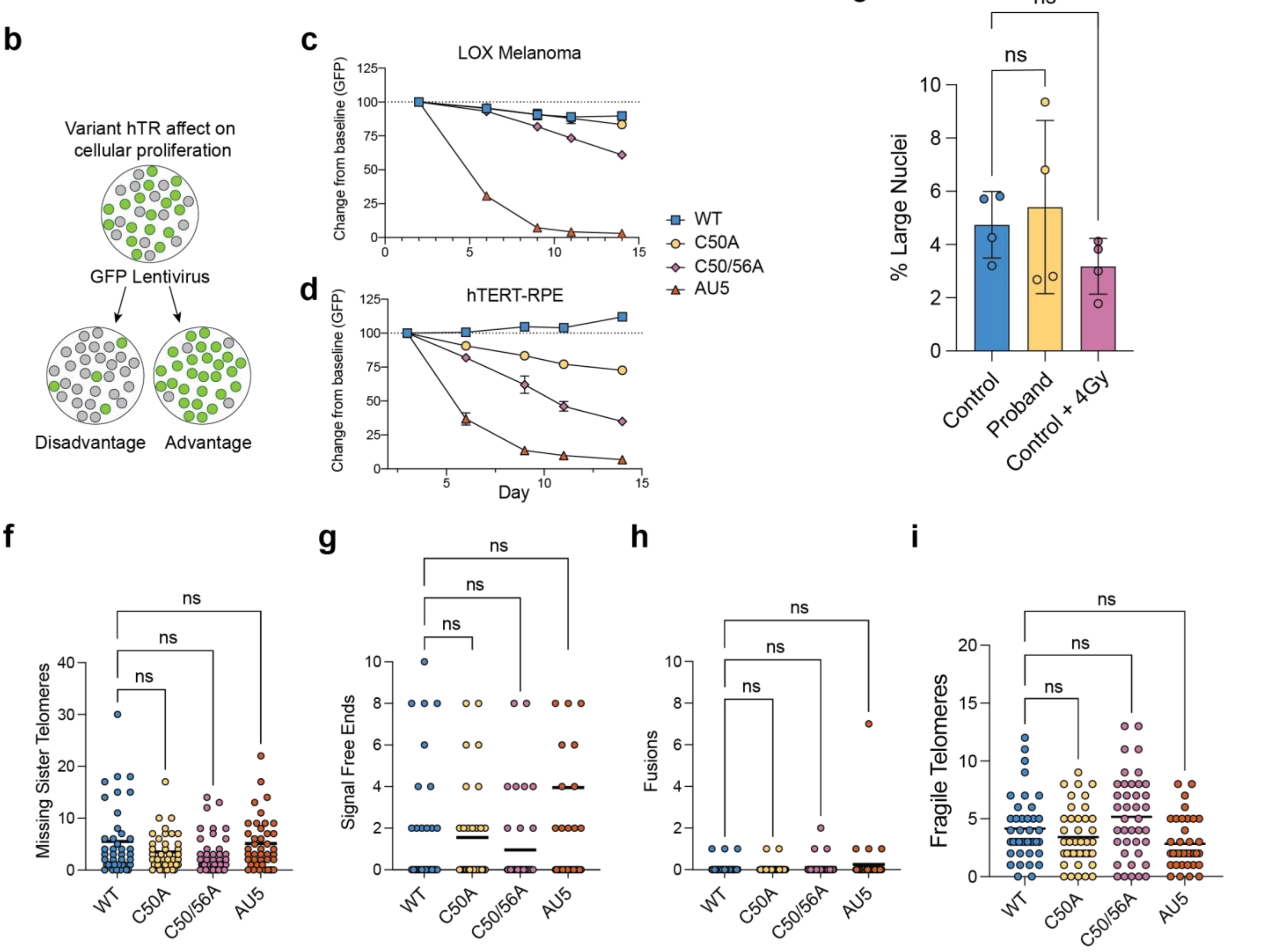

Supplementary Figure 5. DDR and chromosomal abnormalities in cells expressing a variant telomere sequence. a) Table displaying the TR variants used and the resulting telomere sequence the encode. **b)** Graphic depicting the competition assay and potential outcomes. **c-d**) LOX Melanoma and hTERT-RPE cells in the competition assay over the course of 14 days. Each point in normalized to the proportion of GFP$^+$ 48 hours after transduction. Data are n=3 biological replicates with mean±s.d. shown. **e)** Percentage of abnormally large nuclei (>300 μm^2) in the lymphoblast cell lines from Figure 4g. **f-i)** Quantification of chromosomal abnormalities per metaphase in hTERT-RPE with TR overexpression. Each point is a metaphase, compared by a Kruskal-Wallis test with Dunn's multiple correction. For all comparisons, ns, non-significant, $p \geq 0.05$, $^{**}p < 0.01$, $^{****}p < 0.0001$. Source data are provided as a Source Data file.

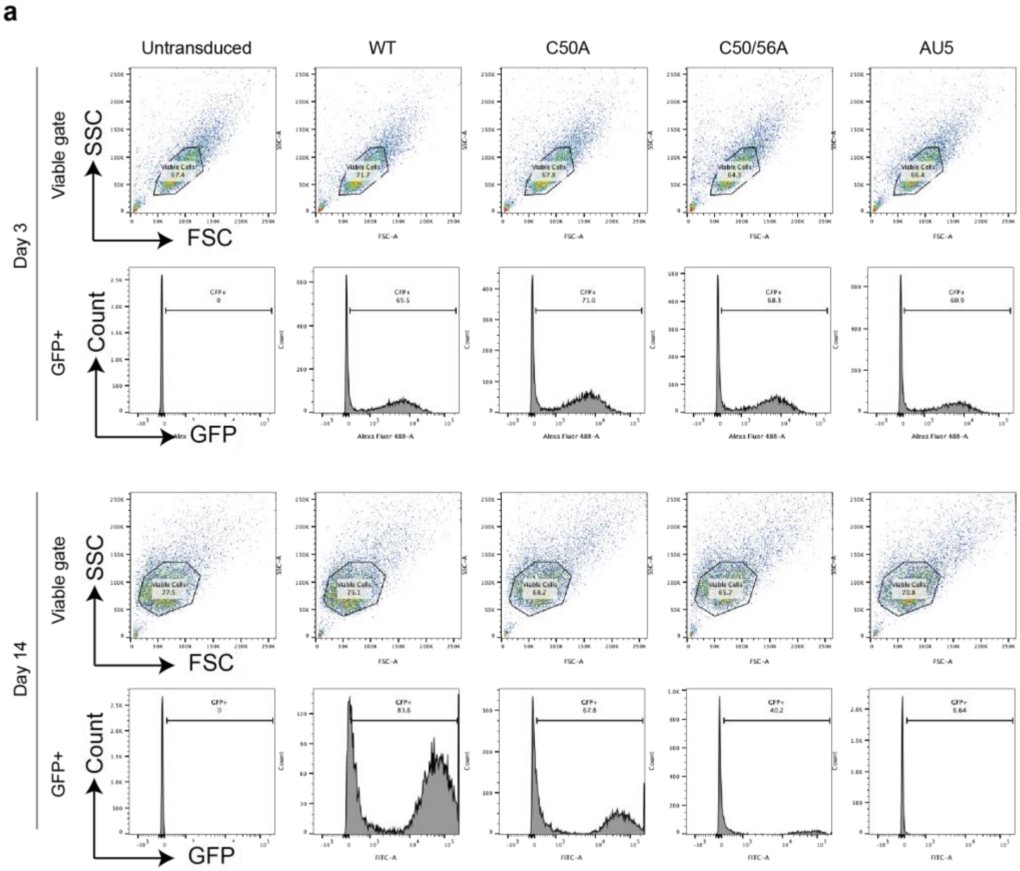

Supplementary Figure 6. Representative flow cytometry data. Flow data from the competition assay described in Extended Data Figure 5b-d. Data is selected from the first and last days of the experiment. Experiment was performed on a Fortessa Flow Cytometer and a minimum of 10,000 data point were collected for each group. **a)** Representative data from hTERT-RPE is shown. Gating strategy of viable and GFP-positive cells are shown. FCS- forward scatter, SSC- side scatter.

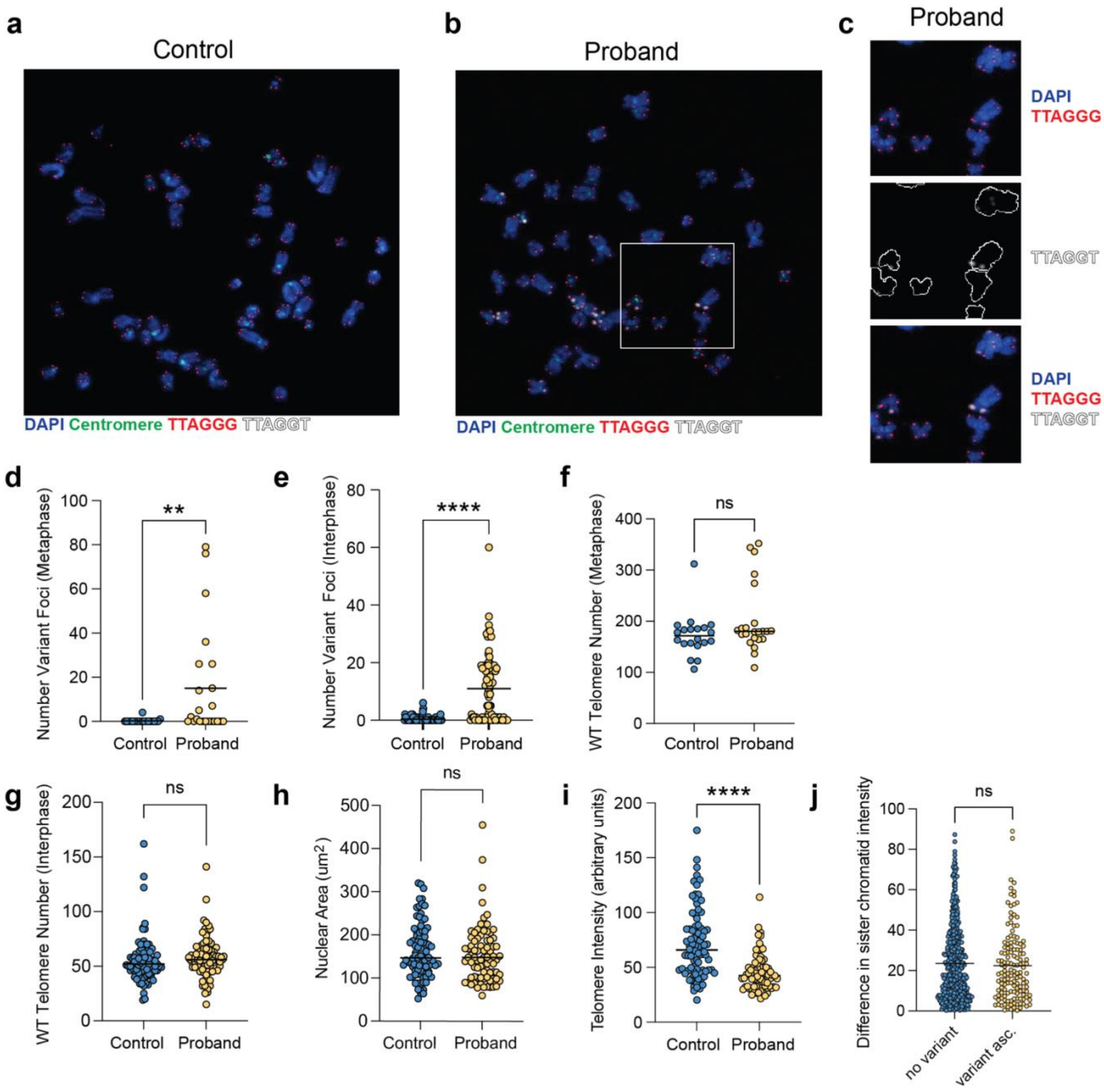

Supplementary Figure 7. Telomere length analysis in patient-derived lymphoblasts. a-b) Representative photomicrographs of metaphase spreads from the control and proband lymphoblast cell lines. **c)** Inset from b, showing the variant telomere on its own and associated with the wildtype telomere. **d-e)** Counts of variant telomere foci in the control and proband lymphoblasts are shown from metaphases (d) and interphase cells (e). **f-g)** Wildtype telomere foci number per metaphases (f) and interphase (g) for control and proband lymphoblasts. **h)** Mean nuclear area is shown for proband and control lymphoblasts. **i)** Measured wildtype telomere intensity by interphase FISH for the control (n=87) and proband (n=79) lymphoblast cell lines. **j)** The difference in sister telomere intensity was calculated for chromosome ends associated or not associated with a variant telomere. Each dot represents the absolute value of the difference between a pair of sister telomeres. Non-variant (n = 435), variant associated (n = 135). For (d-i), the following numbers of interphase cells were analyzed; control (n = 87) and proband (n=79). For metaphase analysis, we examined control (n=20) and proband (n=23). (d-e) and (j) were compared with a Mann-Whitney test and (f-i) were compared with a two-tailed unpaired t-test. For all comparisons, ns, non-significant, $p \geq 0.05$, $^{**}p < 0.01$, and $^{****}p < 0.0001$. Source data are provided as a Source Data file.

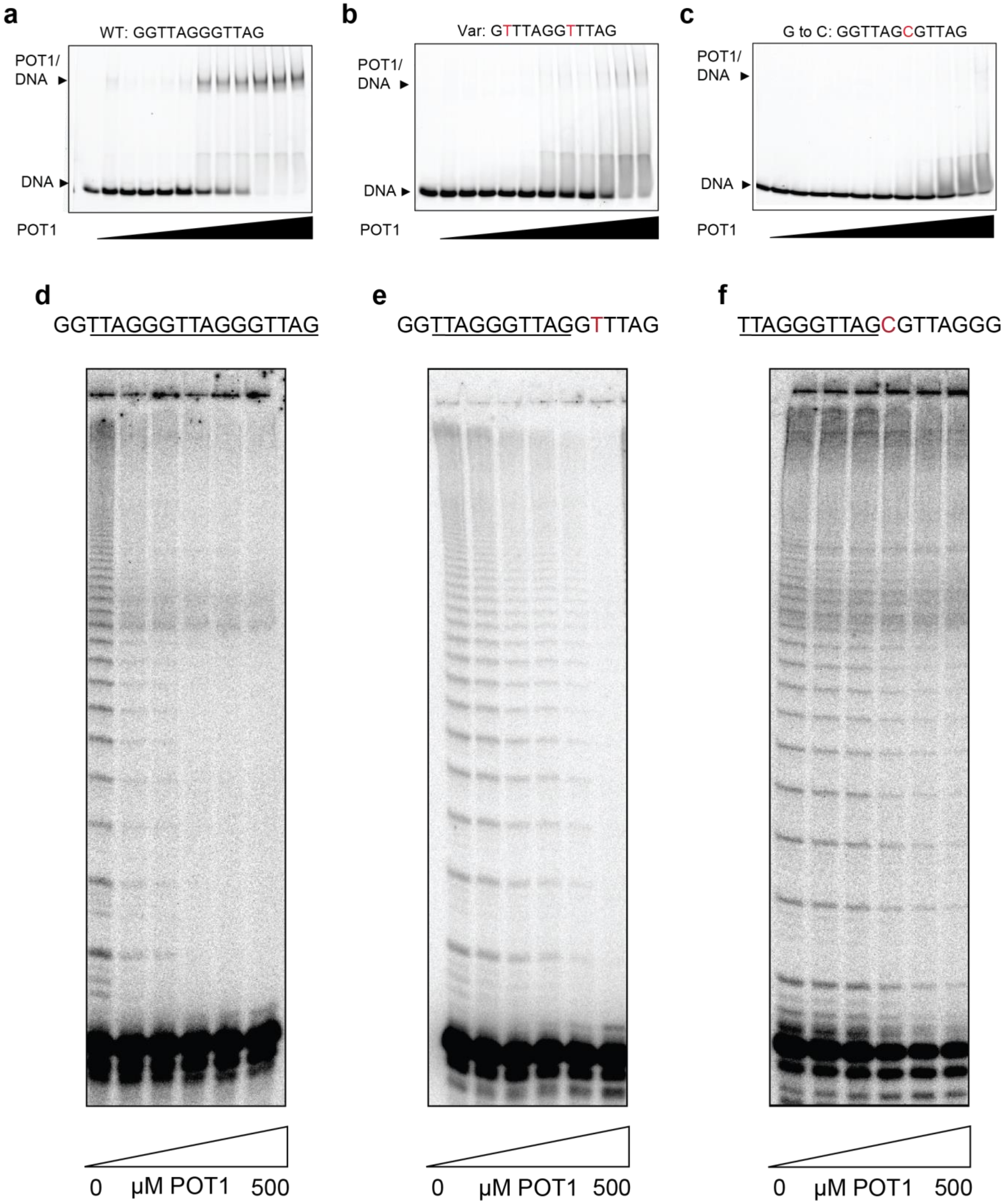

Supplementary Figure 8. POT1 inhibition of telomerase activity is sequence dependent. a-c) Representative images of electromobility shift assays (EMSAs) for POT1 with single-stranded DNA oligonucleotides corresponding to either **a)** the G-strand wildtype telomere sequence (WT), **b)** the variant TTAGGT sequence (C50A), or **c)** a G to C substitution of the wildtype sequence in the POT1 binding site **d-f)**

Representative telomerase direct activity assays with primer pre-incubated with POT1 in concentrations ranging from 0-500μM with 1:2 dilutions. Primers used are **d)** WT, **e)** C50A, and **f)** A5**.** The underline denotes the POT1 binding site. Source data are provided as a Source Data file.

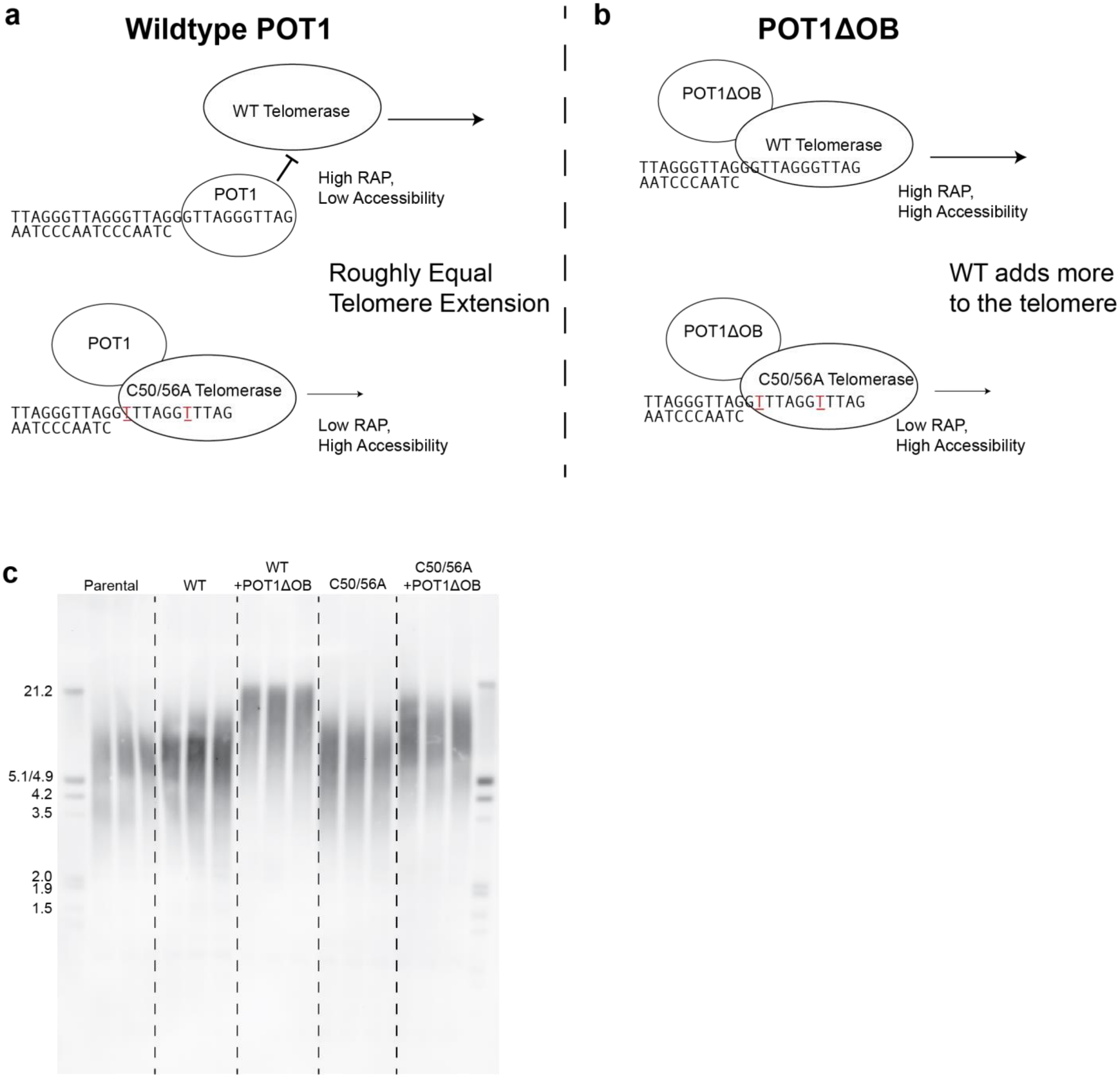

Supplementary Figure 9. TRF Southern Blot with POT1ΔOB. a-b) Models for the different responses to POT1 and POT1ΔOB in cells expressing canonical and non-canonical TRs. **c)** TRF Southern blot quantified in Figure 6 of HCT116 cells 13 days post transduction. Cells were transduced with WT or C50/56A TR and POT1ΔOB.
