# Supplementary material for: A persistent variant telomere sequence in a human pedigree
Source: Nat Commun. 2024 Jun 1;15:4681. doi: 10.1038/s41467-024-49072-9 (PMC11144197; doi:10.1038/s41467-024-49072-9)
Supplement: Supplementary file 3 — Reporting Summary [file 41467_2024_49072_MOESM3_ESM.pdf]

nature portfolio

Corresponding author(s): Jonathan K. Alder

Last updated by author(s): May 17, 2024

Reporting Summary

Nature Portfolio wishes to improve the reproducibility of the work that we publish. This form provides structure for consistency and transparency in reporting. For further information on Nature Portfolio policies, see our Editorial Policies and the Editorial Policy Checklist.

Statistics

For all statistical analyses, confirm that the following items are present in the figure legend, table legend, main text, or Methods section.

n/a	Confirmed	
☐	☒	The exact sample size (*n*) for each experimental group/condition, given as a discrete number and unit of measurement
☐	☒	A statement on whether measurements were taken from distinct samples or whether the same sample was measured repeatedly
☐	☒	The statistical test(s) used AND whether they are one- or two-sided<br>*Only common tests should be described solely by name; describe more complex techniques in the Methods section.*
☒	☐	A description of all covariates tested
☐	☒	A description of any assumptions or corrections, such as tests of normality and adjustment for multiple comparisons
☐	☒	A full description of the statistical parameters including central tendency (e.g. means) or other basic estimates (e.g. regression coefficient) AND variation (e.g. standard deviation) or associated estimates of uncertainty (e.g. confidence intervals)
☐	☒	For null hypothesis testing, the test statistic (e.g. *F*, *t*, *r*) with confidence intervals, effect sizes, degrees of freedom and *P* value noted<br>*Give P values as exact values whenever suitable.*
☒	☐	For Bayesian analysis, information on the choice of priors and Markov chain Monte Carlo settings
☒	☐	For hierarchical and complex designs, identification of the appropriate level for tests and full reporting of outcomes
☒	☐	Estimates of effect sizes (e.g. Cohen's *d*, Pearson's *r*), indicating how they were calculated

Our web collection on statistics for biologists contains articles on many of the points above.

Software and code

Policy information about availability of computer code

Data collection	Microscopy: Nikon Elements Version 5.30.01<br>Southern blotting: Bio-Rad Image Lab Touch Verions 2.3.0.07<br>Flow Cytometry: BD FACSDiva Software
Data analysis	Microscopy: Nikon Elements (GA3) 5.30.01 and ImageJ version 2.1.0/1.53c<br>Southern Blot analysis: Web-based Analyzer of Length of Telomere (WALTER)<br>Flow cytometry analysis:FlowJo Version 10.8.2<br>RNA seq alignment: STAR 2.7.11<br>Sequence analysis was performed with custom perl scripts that are included in the source data<br>Data analysis and visualization: R studio

For manuscripts utilizing custom algorithms or software that are central to the research but not yet described in published literature, software must be made available to editors and reviewers. We strongly encourage code deposition in a community repository (e.g. GitHub). See the Nature Portfolio guidelines for submitting code & software for further information.

Data

Policy information about availability of data

All manuscripts must include a data availability statement. This statement should provide the following information, where applicable:

- Accession codes, unique identifiers, or web links for publicly available datasets
- A description of any restrictions on data availability
- For clinical datasets or third party data, please ensure that the statement adheres to our policy

A subset of the sequencing data generated in this study have been included in the Source data. The dataset contains only the telomeric reads to protect the privacy of the individuals included in this study. The raw whole genome sequencing data are protected and are not available due to data privacy laws. All other source data in this study are provided in the Supplementary Information and Source Data file.

Research involving human participants, their data, or biological material

Policy information about studies with human participants or human data. See also policy information about sex, gender (identity/presentation), and sexual orientation and race, ethnicity and racism.

Reporting on sex and gender	The current study is limited to a single pedigree, limiting the reach of this study for a gender inclusive study. No individuals were excluded based on sex or gender.
Reporting on race, ethnicity, or other socially relevant groupings	Patients undergoing evaluation for lung transplantation and their family members were recruited to this study. No individuals were excluded based on race, ethnicity, or other social groupings.
Population characteristics	The individuals studied here were two members of the same family. The proband described was in his 40s and diagnosed with idiopathic pulmonary fibrosis. The other individual is a 15 year-old child of the proband reported no significant medical concerns.
Recruitment	Individuals with a diagnosis of idiopathic pulmonary fibrosis and who were under evaluation for lung transplantation were recruited to the study. Individuals who agreed to participate in the study were requested to invite their family members to participate.
Ethics oversight	All studies included here were approved by the University of Pittsburgh Institutional Review Board (STUDY 20060250: Lung Transplant Registry & Sample Repository and STUDY 1807008: IPF Genetics Study). All research subjects gave written informed consent and minors assented to study participation.

Note that full information on the approval of the study protocol must also be provided in the manuscript.

Field-specific reporting

Please select the one below that is the best fit for your research. If you are not sure, read the appropriate sections before making your selection.

☒ Life sciences ☐ Behavioural & social sciences ☐ Ecological, evolutionary & environmental sciences

For a reference copy of the document with all sections, see nature.com/documents/nr-reporting-summary-flat.pdf

Life sciences study design

All studies must disclose on these points even when the disclosure is negative.

Sample size	Sample sizes were chosen based on standards in the field and our own experimental experience to obtain reliable results. At least three biologic replicates were performed for all experiments unless otherwise stated.
Data exclusions	No data was excluded in this study.
Replication	Experiments had independent biological replicates, replicated at least three times with the exception of onesouthern blot, which had two total biological replicates due to technical difficulties with samples, and the metaphase spreads in which a minimum of 3,000-6,000 telomere ends were analyzed (~20 - 40 metaphase spreads). The metaphase spreads used for chromosomal anomaly experiments came from three independently transduced populations of cells
Randomization	The control genomes for measuring telomere composition for whole genome sequencing were chosen by choosing 10 sequential numbers of an otherwise randomized and blinded group. No other randomization was necessary for experiments in this manuscript as they did not involve animals experiments or human subjects. Independently transduced wells came from the same parental cell line, minimizing any covariants.
Blinding	Image analysis was performed blinded when possible. When blinding was not possible, images were captured with idential settings and analyzed automated script to minimize bias.

Reporting for specific materials, systems and methods

We require information from authors about some types of materials, experimental systems and methods used in many studies. Here, indicate whether each material, system or method listed is relevant to your study. If you are not sure if a list item applies to your research, read the appropriate section before selecting a response.

Materials & experimental systems

n/a	Involved in the study
☐	☒ Antibodies
☐	☒ Eukaryotic cell lines
☒	☐ Palaeontology and archaeology
☒	☐ Animals and other organisms
☐	☒ Clinical data
☒	☐ Dual use research of concern
☒	☐ Plants

Methods

n/a	Involved in the study
☒	☐ ChIP-seq
☐	☒ Flow cytometry
☒	☐ MRI-based neuroimaging

Antibodies

Antibodies used	Primary Antibodies<br>53BP1 Novus Bio nb100-304 (1:400 for IF)<br>γH2AX Santa Cruz sc517348 (1:400 for IF)<br>Secondar Antibodies<br>Goat anti-Rabbit IgG Alexa Fluor™ 647 A27040 (Invitrogen) (1:1000 for IF)<br>Goat anti-Mouse IgG Alexa Fluor™ 647 A21235(Invitrogen) (1:1000 for IF)
Validation	Antibodies are widely used and were validated using positive controls (gamma-irradiation).

Eukaryotic cell lines

Policy information about cell lines and Sex and Gender in Research

Cell line source(s)	hTERT-RPE-1, a gift from Dr. Patricia Opresko<br>LOX Melanoma (LOX-IMVI), a gift from Dr. Bradley Stohr<br>HCT116 (ATCC® CCL-247™)<br>HEK293FT (ThermoFisher; R70007)
Authentication	hTERT-RPE-1, LOX Melanoma, and HCT116 cell lines were authenticated by ATCC STR profiling. LOX-IMVI are not avialable at ATCC cell line but their STR profile matched the publicly available STR profile for LOX-IMVI cells on the Millipore Sigma website. HEK293FT were not authenticated.
Mycoplasma contamination	Cells were not tested for mycoplasma contamination.
Commonly misidentified lines<br>(See ICLAC register)	No commonly misidentified lines were used in this study.

Clinical data

Policy information about clinical studies

All manuscripts should comply with the ICMJE guidelines for publication of clinical research and a completed CONSORT checklist must be included with all submissions.

Clinical trial registration	N/A
Study protocol	N/A
Data collection	Clinical bio-specimens and data were collected as part of the Lung Transplant Registry & Sample Repository (STUDY 20060250) and and IPF Genetics Study (STUDY 1807008).
Outcomes	This is a observational and bio-repository study.

Plants

Seed stocks	*Report on the source of all seed stocks or other plant material used. If applicable, state the seed stock centre and catalogue number. If plant specimens were collected from the field, describe the collection location, date and sampling procedures.*
Novel plant genotypes	*Describe the methods by which all novel plant genotypes were produced. This includes those generated by transgenic approaches, gene editing, chemical/radiation-based mutagenesis and hybridization. For transgenic lines, describe the transformation method, the number of independent lines analyzed and the generation upon which experiments were performed. For gene-edited lines, describe the editor used, the endogenous sequence targeted for editing, the targeting guide RNA sequence (if applicable) and how the editor was applied.*
Authentication	*Describe any authentication procedures for each seed stock used or novel genotype generated. Describe any experiments used to assess the effect of a mutation and, where applicable, how potential secondary effects (e.g. second site T-DNA insertions, mosiacism, off-target gene editing) were examined.*

Flow Cytometry

Plots

Confirm that:

☒ The axis labels state the marker and fluorochrome used (e.g. CD4-FITC).

☒ The axis scales are clearly visible. Include numbers along axes only for bottom left plot of group (a 'group' is an analysis of identical markers).

☒ All plots are contour plots with outliers or pseudocolor plots.

☒ A numerical value for number of cells or percentage (with statistics) is provided.

Methodology

Sample preparation	Cells were detached using trypsin, trypsin stopped with additional media. Cells were centrifuged at 500xG for 5 minutes and the pellet resuspended in PBS. No antibodies were used, transduced cells expressed GFP
Instrument	BD Fortessa
Software	Flow Cytometry Data was collected using BD FACSDiva Software and analyzed using FlowJo Version 10.8.2
Cell population abundance	No sorting, all cells were used. 10,000 cells were used per group.
Gating strategy	Cells were gated to determine live cells/exclude debris using FSC-A and SCC-A. Cells were gated for GFP positivity using the FITC measurement and gated using untransduced cells as a baseline.

☒ Tick this box to confirm that a figure exemplifying the gating strategy is provided in the Supplementary Information.
